# Supplementary material for: Extracellular matrix-inducing Sox9 promotes both basal progenitor proliferation and gliogenesis in developing neocortex
Source: eLife. 2020 Mar 19;9:e49808. doi: 10.7554/eLife.49808 (PMC7105383; doi:10.7554/eLife.49808)
Supplement: Supplementary file 4. [file elife-49808-supp4.docx]

| **Key Resources Table** | | | | |
| --- | --- | --- | --- | --- |
| **Reagent type (species) or resource** | **Designation** | **Source or reference** | **Identifiers** | **Additional information** |
| Antibody | Rabbit polyclonal anti-Sox9 | Sigma-Aldrich | HPA001758, RRID:AB_1080067 | 1:300 |
| Antibody | Rat monoclonal anti-RFP | ChromoTek | 5F8, RRID:AB_2336064 | 1:500 |
| Antibody | Chicken polyclonal anti-GFP | Aves Labs | GFP-1020,  RRID:AB_10000240 | 1:500 |
| Antibody | Rabbit polyclonal anti-Ki67 | Abcam | ab15580, RRID:AB_443209 | 1:200 |
| Antibody | Mouse monoclonal anti-PCNA | Millipore | CBL407, RRID:AB_93501 | 1:300 |
| Antibody | Goat polyclonal anti-Sox2 | Santa Cruz Biotechnology | sc-17320,  RRID:AB_2286684 | 1:200 |
| Antibody | Rabbit polyclonal anti-Tbr2 | Abcam | ab23345, RRID:AB_778267 | 1:200 |
| Antibody | Sheep polyclonal anti-Tbr2 | R and D Systems | AF6166, RRID:AB_10569705 | 1:500 |
| Antibody | Mouse monoclonal anti-Olig2 | Millipore | MABN50, RRID:AB_10807410 | 1:200 |
| Antibody | Mouse monoclonal anti-NeuN | Millipore | MAB377, RRID:AB_2298772 | 1:200 |
| Antibody | Rat monoclonal anti-phosphohistone H3 | Abcam | ab10543, RRID:AB_2295065 | 1:500 |
| Antibody | Mouse monoclonal anti-phosphovimentin | Abcam | ab22651, RRID:AB_447222 | 1:300 |
| Antibody | Mouse monoclonal anti-Satb2 | Abcam | ab51502, RRID:AB_882455 | 1:300 |
| Antibody | Rabbit monoclonal anti-Tbr1 | Abcam | ab31940, RRID:AB_2200219 | 1:200 |
| Antibody | Rabbit polyclonal anti-Pax6 | Covance | PRB-278P, RRID:AB_291612 | 1:200 |
| Antibody | Goat polyclonal anti- Rabbit Alexa Fluor 405 | ThermoFisher Scientific | A31556, RRID:AB_221605 | 1:500 |
| Antibody | Donkey polyclonal anti- Rabbit Alexa Fluor 488 | ThermoFisher Scientific | A21206,  RRID:AB_141708 | 1:500 |
| Antibody | Goat polyclonal anti- Chicken Alexa Fluor 488 | ThermoFisher Scientific | A11039,  RRID:AB_142924 | 1:500 |
| Antibody | Donkey polyclonal anti-Mouse  Alexa Fluor 555 | ThermoFisher Scientific | A31570,  RRID:AB_2536180 | 1:500 |
| Antibody | Goat polyclonal anti-Rat  Alexa Fluor 555 | ThermoFisher Scientific | A21434, RRID:AB_141733 | 1:500 |
| Antibody | Donkey polyclonal anti-Sheep  Alexa Fluor 555 | ThermoFisher Scientific | A-21436, RRID:AB_2535857 | 1:500 |
| Antibody | Donkey polyclonal anti-Rabbit  Alexa Fluor 647 | ThermoFisher Scientific | A-31573,  RRID:AB_2536183 | 1:500 |
| Antibody | Goat polyclonal anti-Rat Alexa  Fluor 647 | ThermoFisher Scientific | A-21247, RRID:AB_141778 | 1:500 |
| Antibody | Donkey polyclonal anti-Goat Alexa Fluor 647 | ThermoFisher Scientific | A21447,  RRID:AB_141844 | 1:500 |
| Antibody | Donkey polyclonal anti-Mouse Alexa Fluor 647 | ThermoFisher Scientific | A31571,  RRID:AB_162542 | 1:500 |
| Biological sample (*Homo Sapiens*) | Human fetal brain tissue | Klinik und Poliklinik für Frauenheilkunde und Geburtshilfe, Universitätsklinikum Carl Gustav Carus of the Technische Universität Dresden |  |  |
| Biological sample (*Homo Sapiens*) | Human fetal brain tissue | Novogenix Laboratories |  |  |
| Commercial assay or kit | Click-iT EdU Alexa Fluor 647  Imaging Kit | Invitrogen | C10340 |  |
| Commercial assay or kit | Maxi Prep Kit | Qiagen | Cat#12362 |  |
| Commercial assay or kit | RNeasy Micro Kit | Qiagen | Cat#74004 |  |
| Commercial assay or kit | MACS Neural Tissue Dissociation kit (P) | Miltenyi Biotec | Cat#130-092-628 |  |
| Peptide, recombinant protein | Human recombinant laminin 211 | BioLamina | LN211 | 0.1 mg/ml |
| Peptide, recombinant protein | Native human Collagen IV (FAM) | Abcam | ab123531 | 0.1 mg/ml |
| Recombinant DNA reagent | pCAGGS-LoxP-membraneGAP43-GFP-LoxP-IRES-nRFP | Wong et al., 2015 |  |  |
| Recombinant DNA reagent | pCAGGS-LoxP-membraneGAP43-GFP-LoxP-Sox9-IRES-nRFP | This paper |  | See Materials and Methods section, and Fig. 4 |
| Recombinant DNA reagent | FUW-TetO-Sox9 plasmid | Addgene | Cat#41080 |  |
| Recombinant DNA reagent | LacZ CRISPR/Cas9 plasmid | Kalebic et al., 2016 |  |  |
| Recombinant DNA reagent | Sox9 KO CRISPR/Cas9 plasmid | This paper |  | See Materials and Methods section, and Fig. 3 Supplement 1 |
| Sequence-based reagent | **Sox9-F** AGGAAGCTGGCAGACCAGT | This paper |  | Oligonucleotide |
| Sequence-based reagent | **Sox9-R** CTCCTCCACGAAGGGTCTCT | This paper |  | Oligonucleotide |
| Sequence-based reagent | **Olig2-F:** CCCCAGGGATGATCTAAGC | This paper |  | Oligonucleotide |
| Sequence-based reagent | **Olig2-R:** CAGAGCCAGGTTCTCCTCC | This paper |  | Oligonucleotide |
| Sequence-based reagent | **Tbr2-F:** GACCTCCAGGGACAATCTGA | This paper |  | Oligonucleotide |
| Sequence-based reagent | **Tbr2-R:** GTGACGGCCTACCAAAACAC | This paper |  | Oligonucleotide |
| Sequence-based reagent | **Gapdh-F:** TGAAGCAGGCATCTGAGGG | This paper |  | Oligonucleotide |
| Sequence-based reagent | **Gapdh-R:** CGAAGGTGGAAGAGTGGGAG | This paper |  | Oligonucleotide |
| Software, algorithm | Prism | GraphPad |  |  |
| Software, algorithm | ZEN | Carl Zeiss |  |  |
| Software, algorithm | Fiji/ImageJ | Fiji/ImageJ |  | https://imagej.net/Fiji |
| Software, algorithm | R Software | R project |  | https://www.r-project.org/ |
| Software, algorithm | DAVID | Laboratory of Immunopathogenesis and Bioinformatics |  | https://david.ncifcrf.gov/ |
| Software, algorithm | Enrichr | Ma'ayan Lab |  | https://amp.pharm.mssm.edu/Enrichr/ |
| Strain,  strain background  (*Mustela*  *putorius furo*) | Ferret | Marshall  Bioresources |  |  |
| Strain,  strain background  (*Mustela*  *putorius furo*) | Ferret | EuroFerret |  |  |
| Strain,  strain background  (*Mus Musculus*) | *Tis21*-CreER^T2^ transgenic mice | Wong et al., 2015 |  |  |
| Strain,  strain background  (*Mus Musculus*) | C57BL/6JOlaHsd | Envigo |  |  |
